# De-Glycyrrhizinated Licorice Extract Attenuates High Glucose-Stimulated Renal Tubular Epithelial–Mesenchymal Transition via Suppressing the Notch2 Signaling Pathway

**DOI:** 10.3390/cells9010125

**Published:** 2020-01-05

**Authors:** Yung-Chien Hsu, Pey-Jium Chang, Chun-Wu Tung, Ya-Hsueh Shih, Wen-Chiu Ni, Yi-Chen Li, Takuhiro Uto, Yukihiro Shoyama, Cheng Ho, Chun-Liang Lin

**Affiliations:** 1 Departments of Nephrology, Chang Gung Memorial Hospital, Chiayi 613, Taiwan; libra@cgmh.org.tw (Y.-C.H.); peyjiumc@mail.cgu.edu.tw (P.-J.C.); P122219@cgmh.org.tw (C.-W.T.); rita1608@gmail.com (Y.-H.S.); chu_chu73@yahoo.com.tw (W.-C.N.); kame769@cgmh.org.tw (Y.-C.L.); 2 Kidney and Diabetic Complications Research Team (KDCRT), Chang Gung Memorial Hospital, Chiayi 613, Taiwan; 3 Graduate Institute of Clinical Medical Sciences, College of Medicine, Chang Gung University, Taoyuan 333, Taiwan; 4 Faculty of Pharmaceutical Science, Nagasaki International University, 2825-7 Huis Ten Bosch, Sasebo, Nagasaki 859-3298, Japan; uto@niu.ac.jp (T.U.); shoyama@niu.ac.jp (Y.S.); 5 Division of Endocrinology and Metabolism, Chang Gung Memorial Hospital, Chiayi 613, Taiwan; 6 Kidney Research Center, Chang Gung Memorial Hospital, Taipei 105, Taiwan; 7 School of Traditional Chinese Medicine, College of Medicine, Chang Gung University, Taoyuan 333, Taiwan

**Keywords:** diabetic nephropathy, renal fibrosis, renal tubular epithelial cells, EMT, notch2, licorice, glycyrrhizin, de-glycyrrhizinated licorice

## Abstract

Tubulointerstitial fibrosis is a major pathological hallmark of diabetic nephropathy. Increasing evidence has shown that epithelial-to-mesenchymal transition (EMT) of renal proximal tubular cells plays a crucial role in tubulointerstitial fibrosis. Herein, we aimed to elucidate the detailed mechanism of EMT in renal tubular cells under high glucose (HG) conditions, and to investigate the potential of licorice, a medicinal herb, to inhibit HG-induced EMT. Our results showed that renal tubular epithelial cells (normal rat kidney cell clone 52E; NRK-52E) exposed to HG resulted in EMT induction characterized by increased fibronectin and α-SMA (alpha-smooth muscle actin) but decreased E-cadherin. Elevated levels of cleaved Notch2, MAML-1 (mastermind-like transcriptional coactivator 1), nicastrin, Jagged-1 and Delta-like 1 were also concomitantly detected in HG-cultured cells. Importantly, pharmacological inhibition, small interfering RNA (siRNA)-mediated depletion or overexpression of the key components of Notch2 signaling in NRK-52E cells supported that the activated Notch2 pathway is essential for tubular EMT. Moreover, we found that licorice extract (LE) with or without glycyrrhizin, one of bioactive components in licorice, effectively blocked HG-triggered EMT in NRK-52E cells, mainly through suppressing the Notch2 pathway. Our findings therefore suggest that Notch2-mediated renal tubular EMT could be a therapeutic target in diabetic nephropathy, and both LE and de-glycyrrhizinated LE could have therapeutic potential to attenuate renal tubular EMT and fibrosis.

## 1. Introduction

Diabetic nephropathy (DN) is the leading cause of end stage renal disease (ESRD) [1]. DN now accounts for approximately 50% of patients with ESRD and the prevalence of diabetes is predicted to rise from 6% to 10% worldwide in the next decade [2]. During the progression of DN, a decline in renal function is strongly associated with the extent of renal fibrosis, especially tubulointerstitial fibrosis [3]. Renal fibrosis is characterized by excessive deposition and accumulation of extracellular matrix, along with increased numbers of activated myofibroblasts and inflammatory cells in kidney parenchyma [3].

Renal tubulointerstitial fibrosis is an extremely complicated and dynamic process in which myofibroblast activation plays a central role in the development of fibrosis [4]. Multiple studies have implicated that myofibroblasts in renal tubulointerstitial fibrosis could derive from a variety of sources including resident fibroblasts, pericytes, circulating hematopoietic or mesenchymal stem cells from bone marrow, endothelial cells, and proximal tubular epithelial cells [4,5]. Although there are controversies about the origins of myofibroblasts in renal tubulointerstitial fibrosis, renal tubular epithelial cell injury is generally recognized as the key contributor to renal fibrosis [4,5]. Upon injuries, conversion of tubular epithelial cells into mesenchymal-like cells, a process known as epithelial-to-mesenchymal transition (EMT) or dedifferentiation, may lead to lose their cell–cell adhesion with diminished adhesion markers such as E-cadherin, and acquire mesenchymal markers like alpha-smooth muscle actin (α-SMA) and fibronectin [6,7,8]. Despite the fact that several signaling mediators (such as transforming growth factor β/Smads, interleukins, hypoxia-induced factor 1, Twist1 and Snail) are implicated in driving EMT of renal tubular epithelial cells [3,9,10,11], the detailed molecular mechanism of renal tubular EMT under diabetic milieu has not been fully determined.

The Notch pathway is an evolutionally conserved cell-to-cell signaling mechanism in all metazoans [12,13]. There are four different Notch receptors (Notch1 to Notch4) and five ligands (Jagged-1, Jagged-2, Delta-like-1, Delta-like-3, and Delta-like-4) in mammals. Notch receptors are single-pass transmembrane proteins with an extracellular domain containing multiple epidermal growth factor-like repeats and an intracellular domain. Upon ligand binding, the Notch receptor on the plasma membrane undergoes a two-step proteolytic cleavage by ADAM (a disintegrin and metalloprotease) and the γ-secretase complex, respectively, consequently resulting in the release of the Notch intracellular domain (NICD) from the plasma membrane. The NICD then enters into the nucleus, where it complexes with the DNA-binding protein CSL (for CBF-1, Su(H), and Lag-1; also known as RBP-Jκ (recombination signal binding protein for immunoglobulin kappa J region)) and the transcriptional coactivator MAML-1 (mastermind-like protein 1) to cooperatively activate the transcription of downstream target genes such as hairy enhancer of split (HES) and HES-related repressor (HEY) family members [12,13]. Several lines of evidence have suggested that aberrant activation of Notch signaling is closely associated with the progression of DN [13,14]. Murea et al. [15] have reported that activation of Notch signaling in renal tubules correlated well with the degree of tubulointerstitial fibrosis and the decline of renal function in patients with chronic kidney disease. Consistent with this notion, Bielesz et al. [16] showed that genetic deletion of the Notch signaling mediator RBP-Jκ in proximal tubular epithelial cells appeared to ameliorate tubulointersititial fibrosis in the mouse model of folic acid-induced nephropathy. They also found that conditional overexpression of activated Notch1 in tubular epithelial cells caused the development of tubulointerstitial fibrosis [16]. Although all these studies have shown that activation of Notch signaling in renal tubular epithelial cells could contribute to tubulointerstitial fibrosis, the authentic Notch receptor that is responsible for the fibrogenic process in diabetic milieu actually remains obscure.

Licorice (*Glycyrrhiza* spp.) is one of the most commonly prescribed herbs used in traditional Chinese medicine and Japanese Kampo medicine, and is often used as a sweetener or a flavoring agent in many food products and soft drinks [17]. A wide range of pharmaceutical functions for licorice have been reported, which include anti-inflammation, anti-ulcer, anti-virus, anti-bacteria, anti-allergy, and many other activities [17,18,19]. Glycyrrhizin (GC; also known as glycyrrhizic acid) is the major sweet-tasting and bioactive component of licorice. Many bioactivities of GC have been reported in vitro and in vivo, such as anti-inflammatory, anti-oxidant, anti-allergic and anti-cancer activities [17,20,21]. Although GC is generally considered as a safe agent, consuming large quantities or long-term use of GC could cause adverse outcomes, such as hypertension, hypokalemia, and edema [22]. In addition to GC, licorice has been proposed to contain other bioactive components, including flavonoids, chalcones, isoflavonoids and coumarins [17,19,21]. In our previous work, we have developed a new method using an anti-GC monoclonal antibody to prepare GC-knockout licorice and have already demonstrated several biological activities of the prepared GC-knockout licorice [23,24]. To avoid the potential adverse effects of GC, de-glycyrrhizinated (or GC-knockout) licorice has currently been manufactured as a herbal supplement, which is used to treat gastric and duodenal ulcers. Until now, the potential benefits of licorice extract (LE) or de-glycyrrhizinated LE in preventing diabetes-induced renal fibrosis has not been determined.

In this study, we aimed to examine the role of the Notch signaling pathway in EMT induction of renal tubular epithelial cells under high glucose (HG) conditions, and to investigate the potential benefits of LE and de-glycyrrhizinated LE in preventing HG-induced tubular EMT. Using NRK-52E (normal rat kidney cell clone 52E) cells as an in vitro model system, we demonstrated that HG treatment induced EMT via activation of the Notch2 signaling pathway. Moreover, we showed that LE was able to inhibit HG-stimulated EMT in NRK-52E cells by suppressing Notch2 signaling. To our surprise, we noticed that de-glycyrrhizinated LE had comparable efficacy to LE in blocking EMT in HG-cultured NRK-52E cells, whereas GC showed little anti-EMT activity. Our findings therefore implicated that both LE or de-glycyrrhizinated LE could have the therapeutic potential to combat renal tubular EMT and fibrosis in DN.

## 2. Materials and Methods

### 2.1. Cell Culture, Reagents and Transfections

NRK-52E cells, a rat renal proximal tubular cell line, were obtained from the American Type Culture Collection (ATCC; #CRL-1571). The cells were cultured in Dulbecco’s modified Eagle’s medium (DMEM) containing 10% fetal bovine serum, 100 U/mL penicillin, and 100 mg/mL streptomycin in an atmosphere of 5% CO_2_ at 37 °C. To mimic the condition of hyperglycemia, NRK-52E cells were cultured in high concentrations of D-glucose (15 mM, 25 mM or 30 mM), and D-mannitol served as an osmotic control for high glucose. GC (Cat #356780, Calbiochem) and RO492907 (Cat #S1575, Selleckchem) were purchased commercially. Transfection experiments were performed using Lipofectamine 2000 reagent according to the manufacturer’s instructions (Thermo Fisher Scientific).

### 2.2. Preparation and Characterization of Licorice Extract and De-Glycyrrhizinated (or GC-Knockout) Licorice Extract

Licorice extracts with or without GC were prepared from licorice root powder (Uchida Wakanyaku Corporation, Tokyo, Japan) as described previously [23,24]. Briefly, the licorice root powder (100 mg) was extracted with methanol (1.2 mL) and filtered. After evaporation with N_2_ gas at 60 °C, the resultant dried extract was dissolved in dimethyl sulfoxide (DMSO). To prepare GC-knockout licorice extract, licorice extract was dissolved in loading buffer (5% methanol), and then applied to an immunoaffinity column that was conjugated with the anti-GC monoclonal antibody. The prepared licorice extracts with or without GC were verified by thin layer chromatography (TLC) with n-BuOH:H_2_O:CH_3_COOH (7:2:1) as the developing solvent, and samples on the TLC were visualized with a UV lamp (254 nm) or 50% H_2_SO_4_ spray reagent. ELISA tests were used to determine the concentrations of GC in licorice extract and GC-knockout licorice extract.

### 2.3. Western Blot Analysis

Western blot analysis was performed as described previously [25]. In brief, protein lysates of cultured cells were prepared using tissue protein extraction reagent (Pierce, Rockford, IL, USA) according to the manufacture’s instruction. Equal amounts of protein lysates were subjected to SDS-polyacrylamide gel electrophoresis. After gel electrophoresis, the separated proteins were transferred onto a polyvinylidene difluoride (PVDF) membrane (Bio-Rad) and were then probed with specific primary antibodies. Primary antibodies against fibronectin (ab45688; Abcam; 1:2000 dilution), α-SMA (ab7817; Abcam; 1:2000 dilution), E-cadherin (#610181; BD Biosciences; 1:2000 dilution), NICD2 (#5732S; Cell Signaling; 1:1000 dilution), NICD1 (#2421S; Cell Signaling; 1:750 dilution), nicastrin (ab68145; Abcam; 1:1000 dilution), MAML-1 (ab65090; Abcam; 1:1000 dilution), Myc-tag (ab9106; Abcam; 1:2000 dilution), Jagged-1 (sc-390177; Santa Cruz; 1:1000 dilution), Delta-like 1 (#2588; Cell Signaling; 1:1000) and β-actin (#4970S; Cell Signaling; 1:6000 dilution) were obtained commercially. Secondary antibodies used for protein detection included goat anti-rabbit IgG-Horseradish Peroxidase (sc-2030; Santa Cruz; 1:3000 dilution) or goat anti-mouse IgG-HRP (sc-2005; Santa Cruz; 1:3000 dilution).

### 2.4. Knockdown of MAML-1 and Overexpression of NICD2

To knock down MAML-1 expression in cultured NRK-52E cells, cells were transfected with a mix of 4 SureSilencing plasmids expressing short hairpin RNAs (shRNAs) (Cat #KH06329G, Qiagen) that specifically target MAML-1 mRNA. Transfection of a control shRNA plasmid was used as a negative control in the experiments. To overexpress NICD2 in NRK-52E cells, the plasmid pCMV-Myc-NICD2 (a gift from Dr. Min-Jen Tseng at National Chung Cheng University, Taiwan) that encodes Myc-tagged NICD2 was transiently transfected into cells.

### 2.5. Measurement of γ-Secretase Activity by Reporter Assay

A luciferase reporter assay was used to quantify the γ-secretase activity in cells as described previously [26]. In these experiments, NRK-52E cells were co-transfected with the Gal4-driven luciferase reporter plasmid (designated Gal4-Luc) and the chimeric plasmid (designated C99-GV) that encodes Gal4/VP16 fused to the C-terminal fragment (C99) of amyloid precursor protein. The membrane-tethered C99 domain of the C99-GV fusion protein served as a direct substrate of γ-secretase. Both Gal-Luc and C99-GV plasmids were gifts from Dr. Yung-Feng Liao (Academia Sinica, Taiwan). Upon cleavage of the C99-GV fusion protein by γ-secretase in cells, the cleaved protein domain containing Gal4/VP16 could be released from the plasma membrane, which consequently activated the Gal4-driven luciferase reporter. At 48 h post-transfection, the luciferase activity in transfected cells was measured according to the manufacturer’s protocol for the luciferase reporter assay system (Promega).

### 2.6. Reporter Assay for Monitoring Notch Signaling Activation

The luciferase reporter plasmid that contains two tandem copies of CSL (or RBP-Jκ)-binding sites from the Hes5 promoter [27] was used for transient transfection in NRK-52E cells. After a 48 h transfection, the luciferase activities in treated cells were assayed according to the manufacturer’s protocol (Promega).

### 2.7. Quantitative Reverse Transcription (RT)-PCR

Extraction of total RNA, reverse transcription, real-time PCR based on SYBR Green I fluorescent dye (*N*,*N*-dimethyl-*N*’-[4-[(E)-(3-methyl-1,3-benzothiazol-2-ylidene)methyl]-1-phenylquinolin-1-ium-2-yl]-*N*’-propylpropane-1,3-diamine), and analysis of quantitative PCR data were performed as mentioned previously [25]. The PCR primers used are following: 5′-GTCTGCAAAGAAGGCTGGGA and 5′-GCCACACCAGACCTTGGAGC for *Jagged-1*; 5′-TGCACACACAACACCAATGA and 5′-CACTGGGCTGAGGGGACAGC for *Jagged-2*; 5′-CAACCCCATCCGATTCCCCT and 5′-GTCACAATATCCATGTTGGT for *Delta-like 1*; 5′-GAAATTCACTTATCAGCCAA and 5′-CAGGGGATGGTGCAGGT for *Delta-like 4*.

### 2.8. Statistical Analysis

All data were expressed as mean ± standard error of mean (SEM). The Wilcoxon two-sample test was used to evaluate differences between samples. Parametric ANOVA and a Bonferroni post hoc test were used to analyze the differences among different treated groups. Statistical analysis was performed using SPSS (Statistical Package for the Social Sciences) version 18.0. *p* < 0.05 was considered statistically significant.

## 3. Results

### 3.1. High Glucose Promotes EMT and Notch2 Activation in Renal Tubular NRK-52E Cells

**Figure 1:**
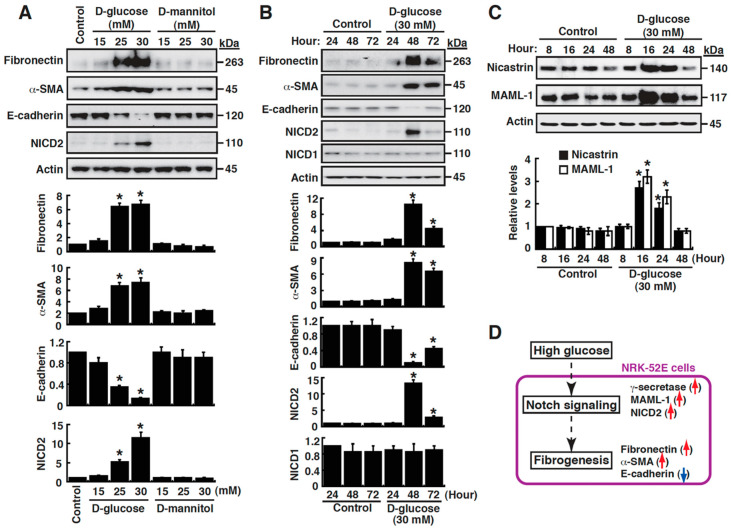
High glucose induces EMT (epithelial-mesenchymal transition) and Notch2 activation in renal tubular epithelial cells. (**A**) Effect of high glucose concentrations on the expression of EMT markers (including fibronectin, alpha-smooth muscle actin (α-SMA) and E-cadherin) and cleaved Notch2 (NICD2) in NRK-52E cells. * *p* < 0.05 versus the normal control (5.5 mM glucose), *n* = 3. (**B**) Time-course effect of high glucose on the promotion of fibrosis and Notch2 activation in NRK-52E cells. * *p* < 0.05 versus normal controls for the indicated time points, *n* = 3. (**C**) Modulation of Notch signaling components including nicastrin and MAML-1 (mastermind-like protein 1) by high glucose at different time points in NRK-52E cells. * *p* < 0.05 versus normal controls for the indicated time points, *n* = 3. (**D**) Proposed roles of Notch2 activation in high glucose-mediated tubular fibrosis.

To investigate the potential molecular mechanism underlying diabetic renal fibrosis, renal proximal tubular epithelial cells (NRK-52E) cultured in normal (5.5 mM) or high glucose (HG; 15 mM, 25 mM and 30 mM) were used as an experimental model. After 48 h of culture, our results showed that HG, but not high mannitol, significantly upregulated the expression of fibronectin and α-SMA, two well-known myofibroblast markers, and downregulated E-cadherin expression in a dose-dependent manner (Figure 1A). Time course experiments also confirmed that increased levels of fibronectin and α-SMA but reduced levels of E-cadherin were detected in HG-cultured NRK-52E cells (Figure 1B). These results indicated that HG was able to induce EMT in renal tubular cells. During the course of the experiments, we additionally found that elevated levels of activated Notch2 (NICD2), but not activated Notch1 (NICD1), were concomitantly detected in HG-cultured NRK-52E cells (Figure 1A,B). Interestingly, when other Notch signaling components were examined, we showed that nicastrin, the largest component of γ-secretase [28], and MAML-1 were remarkably upregulated in NRK-52E cells during the early period (from 16 h to 24 h) of exposure to HG (Figure 1C). According to these results, we hypothesized that Notch2 signaling activation could play a vital role in HG-induced EMT of renal tubular cells, a key process involved in renal fibrogenesis (Figure 1D).

### 3.2. Effects of Licorice Extract (LE), Glycyrrhizin (GC), and GC-Knockout LE on HG-Induced EMT in NRK-52E Cells

**Figure 2:**
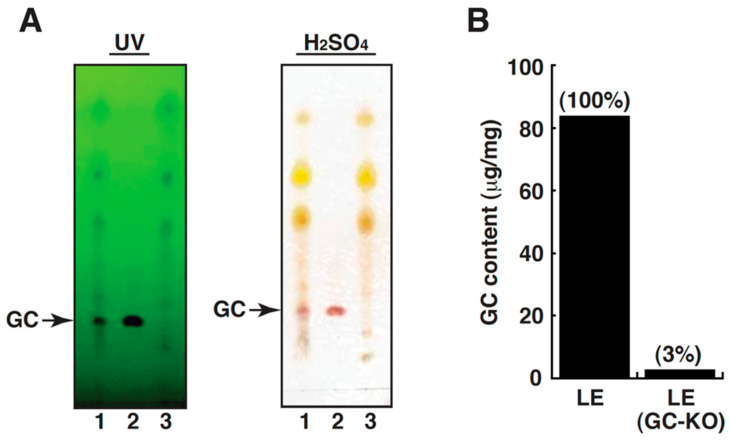
Preparation and characterization of licorice extract (LE) and glycyrrhizin (GC)-free licorice extract (LE/GC-knockout (KO)). (**A**) Thin layer chromatography (TLC) profiling of LE, LE/GC-KO and a GC standard. Lane 1: LE; lane 2: GC; lane 3: LE/GC-KO. Noteworthily, the GC content normally accounts for 7% to 10% of LE. (**B**) ELISA analysis of the GC content in LE and in LE/GC-KO samples.

In order to determine whether LE or GC-knockout LE (designated “LE/GC-KO” in this study) had the ability to inhibit HG-stimulated EMT, we first prepared methanolic LE from licorice root powder. To obtain LE/GC-KO, the immunoaffinity column conjugated with anti-GC monoclonal antibody was used to remove GC from LE. In TLC analysis, we confirmed that the spot of GC was only detected in LE, but not in LE/GC-KO (Figure 2A). Moreover, ELISA measurement of GC revealed that one milligram of LE and LE/GC-KO contained 83.43 μg and 2.53 μg of GC, respectively, indicating that the depletion efficiency of GC by immunoaffinity column was up to 97% (Figure 2B). It is worth mentioning that the prepared LE generally contains approximately 7–10% GC.

**Figure 3:**
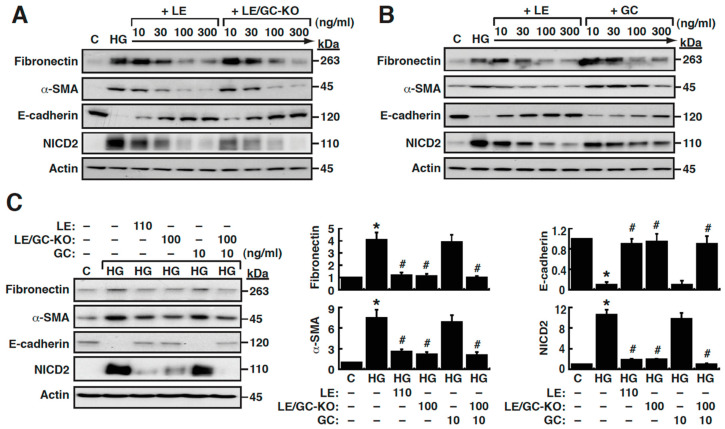
Effects of LE, LE/GC-KO and GC on high glucose (HG)-mediated activation of EMT and Notch2 in NRK-52E cells. (**A**) Comparison of treatment of HG-cultured NRK-52E cells with LE versus LE/GC-KO. Increasing amounts (10 ng/mL, 30 ng/mL, 100 ng/mL and 300 ng/mL) of LE or LE/GC-KO were used to treat HG-cultured NRK-52E cells. At 48 h after treatment, the expression of EMT markers and NICD2 in the treated cells were examined by Western blot analysis. (**B**) Comparison of treatment of HG-cultured NRK-52E cells with LE versus GC. Increasing amounts (10 ng/mL, 30 ng/mL, 100 ng/mL and 300 ng/mL) of LE or GC were used to treat HG-cultured NRK-52E cells. At 48 h after treatment, the expressions of EMT markers and NICD2 in the treated cells were examined by Western blot analysis. (**C**) Changes in the expression levels of EMT markers and NICD2 in HG-cultured NRK-52E cells treated with LE (110 ng/mL), LE/GC-KO (100 ng/mL), GC (10 ng/mL), or the combination of LE/GC-KO (100 ng/mL) and GC (10 ng/mL). At 48 h after treatment, cells were harvested for Western blotting. * *p* < 0.05 versus normal controls, # *p* < 0.05 versus untreated HG-cultured cells (*n* = 3).

Next, increasing amounts of LE, LE/GC-KO or pure GC (10 ng/mL, 30 ng/mL, 100 ng/mL and 300 ng/mL) were used to treat HG-cultured NRK-52E cells. As shown in Figure 3A, LE treatment substantially inhibited HG-induced changes in α-SMA, fibronectin and E-cadherin in a dose-dependent manner. The minimum effective concentration of LE to block EMT induction in HG-cultured NRK-52E cells was around 100 ng/mL. Compared to the anti-EMT effect of LE, we found that LE/GC-KO at the same concentrations had comparable anti-EMT ability in HG-cultured NRK-52E cells (Figure 3A). Noteworthily, increased levels of NICD2 in HG-cultured NRK-52E cells were also remarkably reduced by both LE and LE/GC-KO, suggesting that Notch2 signaling activation was positively associated with HG-induced EMT. On the other hand, although GC is generally considered as the main bioactive component of licorice, our results showed that GC exhibited only weak or moderate anti-EMT activity even at high concentrations (100 ng/mL and 300 ng/mL) (Figure 3B). Moreover, as compared to LE, GC showed little ability to suppress Notch2 activation in HG-cultured NRK-52E cells (Figure 3B).

As mentioned above, GC generally accounts for 7–10% of the prepared LE. To further evaluate the contribution of the GC and non-GC fractions within LE to the inhibition of tubular EMT, NRK-52E cells cultured in HG were treated with LE (110 ng/mL), LE/GC-KO (100 ng/mL), GC (10 ng/mL), or the combination of LE/GC-KO (100 ng/mL) and GC (10 ng/mL). As compared to LE (110 ng/mL) or the combination of LE/GC-KO (100 ng/mL) and GC (10 ng/mL), treatment with LE/GC-KO (100 ng/mL) had a similar anti-EMT ability in HG-cultured NRK-52E cells; however, treatment with GC (10 ng/mL) did not produce detectable anti-EMT ability (Figure 3C). These results suggested that the anti-EMT function of licorice was mainly attributed to the LE/GC-KO fraction rather than GC.

### 3.3. Upregulation of MAML-1 is Critically Required for HG-Mediated EMT in NRK-52E Cells

**Figure 4:**
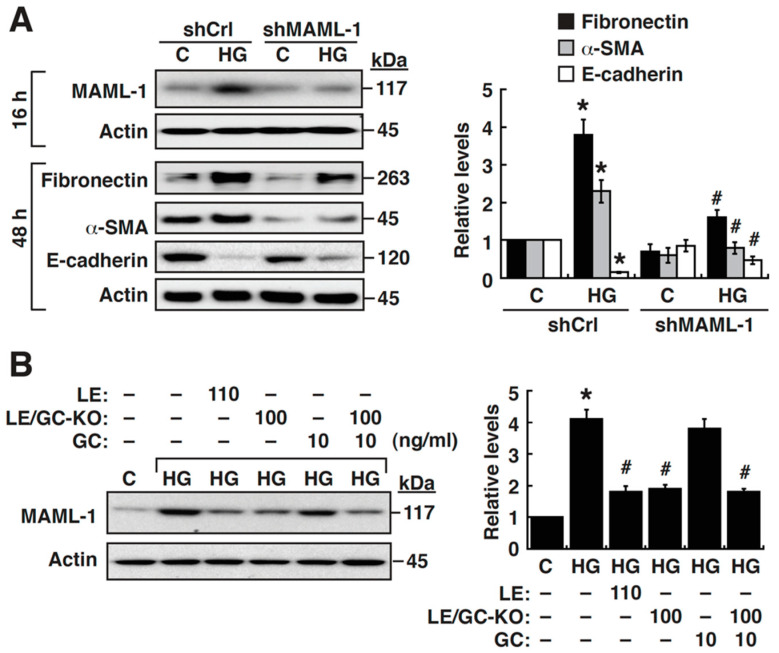
Upregulated expression of MAML-1 is critical for HG-mediated EMT activation in NRK-52E cells, which can be attenuated by LE or LE/GC-KO. (**A**) Suppression of HG-induced EMT in NRK-52E cells by knockdown of MAML-1. Cells were harvested at the indicated time points (16 h or 48 h) after shRNA transfection, and Western blot analysis was performed. * *p* < 0.05 versus the normal control, # *p* < 0.05 versus the control shRNA (short hairpin RNA) group with HG incubation (*n* = 3). (**B**) Changes in MAML-1 expression in HG-cultured NRK-52E cells treated with LE (110 ng/mL), LE/GC-KO (100 ng/mL), GC (10 ng/mL), or the combination of LE/GC-KO (100 ng/mL) and GC (10 ng/mL). At 16 h after treatment, cells were harvested for Western blotting. * *p* < 0.05 versus the normal control, # *p* < 0.05 versus untreated HG-cultured cells (*n* = 3).

In addition to increased levels of NICD2, the Notch co-activator MAML-1 could be also upregulated in HG-cultured NRK-52E cells (Figure 1C). To determine whether the upregulated MAML-1 was involved in the regulation of HG-stimulated EMT in NRK-52E cells, knockdown of MAML-1 by shRNA was performed. As shown in Figure 4A, knockdown of MAML-1 could partially attenuate HG-stimulated EMT in NRK-52E cells. These results suggested that MAML-1 upregulation was critically involved in HG-triggered EMT in NRK-52E cells. Interestingly, when LE (110 ng/mL), LE/GC-KO (100 ng/mL) or GC (10 ng/mL) were used to treat HG-cultured NRK-52E cells, we found that LE (110 ng/mL) or LE/GC-KO (100 ng/mL), but not GC (10 ng/mL), could substantially inhibit MAML-1 upregulation in HG-cultured NRK-52E cells (Figure 4B).

### 3.4. Suppression of γ-Secretase Activity Prevents HG-Triggered EMT in NRK-52E Cells

**Figure 5:**
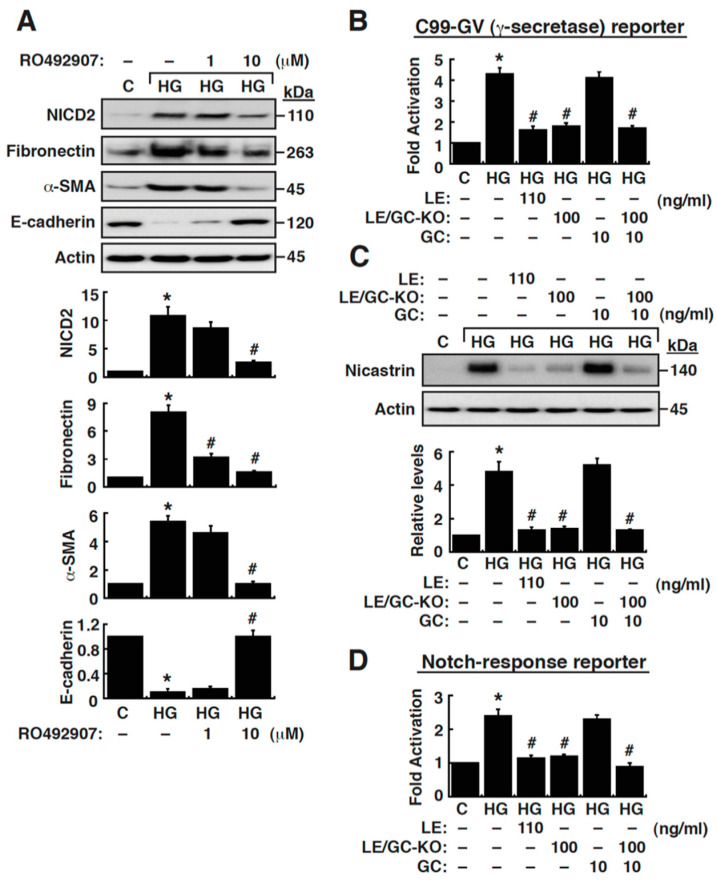
An elevated γ-secretase activity is essential for HG-induced cell fibrosis, which can be blocked by LE or LE/GC-KO. (**A**) Effect of the γ-secretase inhibitor RO492907 on the expression of NICD2 and EMT markers in HG-cultured NRK-52E cells. At 48 h after treatment, cells were harvested for Western blotting. (**B**) Measurement of γ-secretase activity by reporter assay in NRK-52E cells under different treated conditions. Details of the measurement of the γ-secretase activity in treated cells using the Gal4-driven luciferase reporter plasmid and the C99-GV expression plasmid were described in Materials and Methods. At 48 h after transfection and treatment, cells were lysed, and luciferase assays were performed. (**C**) Changes in the expression of nicastrin in HG-cultured NRK-52E cells treated with LE, LE/GC-KO or GC at the indicated concentrations. At 16 h after treatment, cells were harvested for Western blotting. (**D**) Evaluation of activation of a Notch-response reporter construct (Hes5) in NRK-52E cells under different treated conditions. At 48 h after transfection and treatment, cells were lysed, and luciferase assays were performed. * *p* < 0.05 versus the normal control, # *p* < 0.05 versus untreated HG-cultured cells (*n* = 3).

Since activation of Notch2 signaling was closely associated with HG-mediated EMT in NRK-52E cells, the action of γ-secretase in HG-mediated EMT was further evaluated. As expected, treatment of HG-cultured NRK-52E cells with the γ-secretase inhibitor RO492907 resulted in significantly reduced NICD2 levels (Figure 5A). Treatment with RO492907 also reduced the EMT phenotypes showing changes in levels of fibronectin, α-SMA and E-cadherin in HG-cultured NRK-52E cells (Figure 5A). These results strongly suggested that Notch signaling activation was essential for EMT induced by HG in NRK-52E cells. To specifically examine the activity of γ-secretase in NRK-52E cells under different treatment conditions, the γ-secretase activity was assayed using a Gal4-driven luciferase reporter coupled with the plasmid expressing the chimeric C99-GV protein [26]. The C99-GV protein contains the C-terminal fragment of amyloid precursor protein (C99) and the Gal4/VP16 domain, which serves as an immediate substrate of γ-secretase in the assay system. Specific cleavage of C99-GV by γ-secretase would cause the release of the Gal4/VP16 domain from the plasma membrane, thereby activating the Gal4-driven reporter gene expression [26]. Transient transfection analysis showed that HG significantly activated Gal4-driven reporter at high levels in NRK-52E cells (Figure 5B). Using this assay system, we additionally found that the elevated γ-secretase activity in HG-cultured NRK-52E cells could be attenuated by LE (110 ng/mL) and LE/GC-KO (100 ng/mL), but not by GC (10 ng/mL) (Figure 5B).

Due to the observation that HG treatment led to upregulation of nicastrin in NRK-52E cells (Figure 1C and Figure 5C), it was possible that the upregulated expression of nicastrin in HG-cultured NRK-52E cells might at least in part contribute to increased γ-secretase activity. We therefore examined whether treatment of HG-cultured NRK-52E cells with LE, LE/GC-KO or GC altered the expression of nicastrin. Western blot analysis revealed that LE (110 ng/mL) and LE/GC-KO (100 ng/mL), but not GC (10 ng/mL), were able to block the upregulation of nicastrin in HG-cultured NRK-52E cells (Figure 5C). To further assess the Notch signaling cascade, the reporter plasmid that contains Notch-responsive elements from the Hes5 promoter was transiently transfected into NRK-52E cells and the reporter activation was measured. We confirmed that HG treatment enhanced the downstream response of Notch signaling in NRK-52E cells (Figure 5D), and the increased reporter response induced by HG could be inhibited by LE (110 ng/mL) and LE/GC-KO (100 ng/mL), but not by GC (10 ng/mL) (Figure 5D).

### 3.5. Ectopic Expression of NICD2 in NRK-52E Cells Sufficiently Stimulates EMT

**Figure 6:**
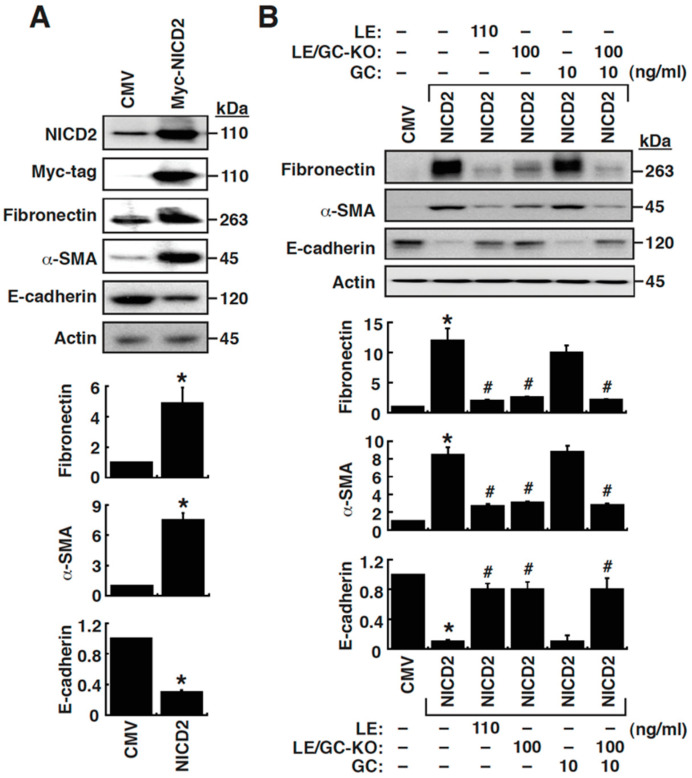
Overexpression of NICD2 is sufficient to induce EMT in NRK-52E cells; however, LE or LE/GC-KO can attenuate NICD2-mediated EMT. (**A**) Activation of EMT in NRK-52E cells by NICD2 overexpression. At 48 h of transfection, cells were harvested for Western blotting. * *p* < 0.05 versus the empty vector control (*n* = 3). (**B**) Effect of LE, LE/GC-KO or GC on NICD2-mediated EMT in NRK-52E cells. At 48 h after transfection and treatment, cells were harvested for Western blotting. * *p* < 0.05 versus the empty vector control, # *p* < 0.05 versus untreated NICD2-transfected control (*n* = 3).

To further determine whether Notch2 activation critically regulated tubular EMT, NICD2 was overexpressed in NRK-52E cells. As shown in Figure 6A, ectopic expression of NICD2 in NRK-52E cells remarkably increased the expression of fibronectin and α-SMA, as well as decreased E-cadherin expression. These results strongly support the importance of Notch2 signaling in EMT of renal tubular cells. To investigate whether LE, LE/GC-KO or GC affected Notch2-mediated EMT in NRK-52E cells, the expression levels of fibronectin, α-SMA and E-cadherin were analyzed by Western blot. Our results showed that Notch2-mediated EMT induction could be substantially attenuated by LE (110 ng/mL) or LE/GC-KO (100 ng/mL), but not GC (10 ng/mL) (Figure 6B).

### 3.6. High Glucose Upregulates the Expression of Jagged-1 and Delta-like 1 in NRK-52E Cells

**Figure 7:**
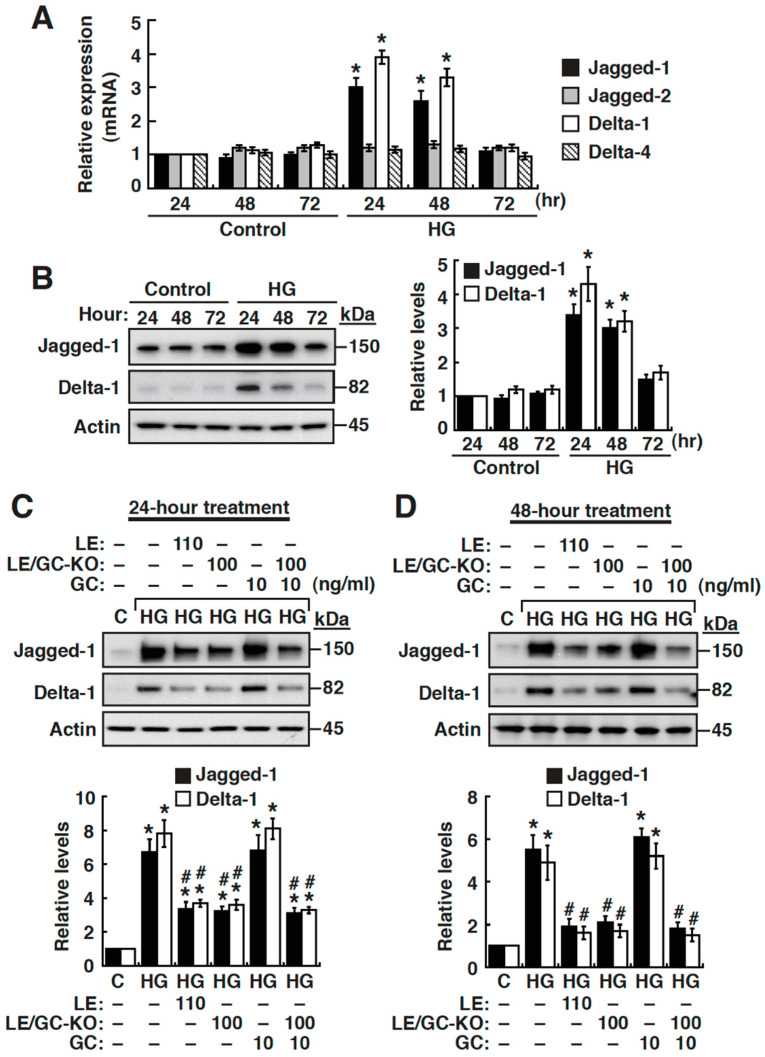
The expression of Jagged-1 and Delta-like 1 is upregulated in HG-cultured NRK-52E cells, which can be attenuated by LE or LE/GC-KO. (**A**) Quantitative RT-PCR analysis of various Notch ligands (including *Jagged-1*, *Jagged-2*, *Delta-like 1* and *Delta-like 4*) in normal or HG-cultured NRK-52E cells. * *p* < 0.05 versus normal controls for the indicated time points, *n* = 3. (**B**) Western blot analysis of Jagged-1 and Delta-like 1 expression in NRK-52E cells cultured in normal or HG conditions. * *p* < 0.05 versus normal controls for the indicated time points, *n* = 3. (**C**,**D**) Effect of LE, LE/GC-KO or GC on the expression of Jagged-1 and Delta-like 1 in HG-cultured NRK-52E cells. At 24 h or 48 h after treatment, cells were harvested for Western blotting. * *p* < 0.05 versus the normal control, # *p* < 0.05 versus untreated HG-cultured cells (*n* = 3).

To search for the potential ligands for Notch2 in HG-cultured cells, we initially examined mRNA levels of *Jagged-1*, *Jagged-2*, *Delta-like 1*, *Delta-like 3* and *Delta-like 4* by quantitative RT-PCR. Our results revealed that NRK-52E cells exposed to HG for 24 h or 48 h produced significantly higher levels of *Jagged-1* and *Delta-like 1* mRNA (with a 3- to 4-fold increase) as compared to normal controls (Figure 7A). However, *Jagged-2* and *Delta-like 4* mRNA did not exhibit significant changes in HG-cultured NRK-52E cells as compared to normal controls (Figure 7A). Exceptionally, under the experimental conditions, *Delta-like 3* mRNA transcripts were very low or undetectable in either normal or HG-cultured NRK-52E cells (data not shown). In addition to quantitative RT-PCR analysis, upregulation of Jagged-1 and Delta-like 1 was also consistently detected in HG-cultured NRK-52E cells by Western blot analysis (Figure 7B). To determine the effects of LE, LE/GC-KO or GC on the expression of Jagged-1 and Delta-like 1 in HG-cultured NRK-52E cells, the 24 h and 48 h treated cells were prepared and subjected to Western blot analysis (Figure 7C,D). The results showed that LE (110 ng/mL) or LE/GC-KO (100 ng/mL), but not GC (10 ng/mL), could significantly attenuate HG-induced upregulation of Jagged-1 and Delta-like 1 in either 24 h treated cells (Figure 7C) or 48 h treated cells (Figure 7D).

## 4. Discussion

**Figure 8:**
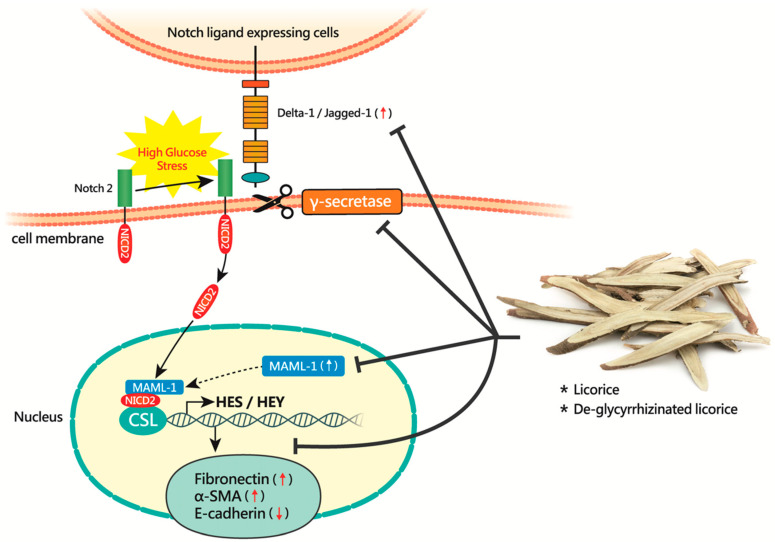
A model for the activation of Notch2 signaling in renal tubular epithelial cells under high glucose conditions and the possible actions of LE or LE/GC-KO in suppressing the Notch2 signaling cascade. Under high glucose conditions, multiple Notch signaling components including Delta-like 1, Jagged-1, cleaved Notch2 (NICD2), nicastrin (a component of the γ-secretase complex), and MAML-1 exhibit marked upregulation in renal tubular epithelial cells. In the nucleus, the activated Notch2 interacting with the transcription factor CSL and the co-activator MAML-1 results in the transcriptional activation of downstream target genes including HES/HEY family members, which are probably required for the modulation of renal tubular EMT and fibrogenesis. As shown in the study, licorice or de-glycyrrhizinated licorice may have different attenuation mechanisms for the activated Notch2 signaling pathway.

Renal tubular EMT is considered as a key process during the development of renal tubulointerstitial fibrosis in DN. Although several signaling pathways or mediators that modulate renal tubular EMT or fibrogenesis in vitro or in vivo have been previously documented, the detailed molecular mechanism of diabetes-induced tubular EMT has not yet been fully understood. Here, we used NRK-52E cells cultured in HG as an in vitro model of DN and demonstrated that Notch2 activation critically contributes to HG-induced EMT. Particularly, our results revealed that both LE and de-glycyrrhizinated LE could prevent NRK-52E cells from HG-induced EMT by targeting Notch2 signaling via multiple different mechanisms (Figure 8). Based on our findings, we believe that LE or de-glycyrrhizinated LE could serve as a potential therapeutic agent for blocking renal tubular EMT and fibrogenesis in DN.

The Notch pathway is well-known to regulate a wide range of cellular processes. Previous studies have shown that an activated Notch pathway is essential for embryonic kidney development [14]. Although the Notch pathway is a critical regulator of kidney development, the expression of most Notch pathway proteins is largely downregulated once kidney development is completed [13,29]. However, when the kidney becomes damaged, the Notch pathway could be reactivated in kidney cells including tubular epithelial cells. Although previous studies have shown that Notch1 expression in renal tubules correlated with tubulointerstitial fibrosis and decreased kidney function in patients with chronic kidney disease [15,16], the role of Notch1 in renal tubular epithelial de-differentiation (or EMT) and in tubulointerstitial fibrosis still remains controversial. Particularly, a more recent study from Huang et al. [30] pointed out that the expression levels of Notch2 and its ligand Jagged-1 substantially correlated with the degree of interstitial fibrosis in several different kidney fibrosis models including folic acid (FA)-induced nephropathy, unilateral obstruction (UUO), or apolipoprotein L1 (APOL1)-associated kidney disease. Furthermore, they found that mice with tubule-specific genetic deletion of Notch2, but not Notch1, conferred protection against FA-induced kidney fibrosis [30]. Consistent with the findings from Huang et al. [30], our data clearly demonstrated that activated Notch2, but not Notch1, was associated with EMT induction in HG-cultured NRK-52E cells (Figure 1 and Figure 6). Besides the increased levels of NICD2, we also found that some other Notch signaling components including MAML-1, nicastrin, Jagged-1 and Delta-like 1 could be also upregulated in HG-cultured NRK-52E cells (Figure 1 and Figure 7). Importantly, siRNA depletion of MAML1 or pharmacological inhibition of the γ-secretase complex significantly attenuated EMT in HG-cultured NRK-52E cells, whereas overexpression of NICD2 sufficiently triggered EMT in NRK-52E cells (Figure 4, Figure 5 and Figure 6). All of our experiments strongly conclude that activation of the Notch2 pathway is important for renal tubular EMT. Additionally, according to our studies and other previous reports [30,31], it may be possible that under hyperglycemic conditions, activation of the Notch signaling pathway in renal tubular epithelial cells could be achieved through multiple ways, including upregulation of Notch2, its ligands including Jagged-1 and Delta-like 1, the γ-secretase component nicastrin, and the transcriptional co-activator MAML-1 (Figure 8). To prevent the progression of renal fibrosis in diabetic milieu, blocking the Notch2 signaling cascade in renal tubular epithelial cells may serve as a promising therapeutic strategy.

Although pharmacological targeting of Notch signaling has been increasingly developed (e.g., γ-secretase inhibitors) and is being applied to clinical trials for cancers or Alzheimer’s disease, most of these drugs have had several significant side effects, especially gastrointestinal toxicities [32,33,34,35]. Due to the concern regarding toxicities of pan-Notch inhibitors, finding a new less toxic agent against Notch signaling pathway may be important not only for cancers but also for specific human disease such as renal tubulointerstitial fibrosis. Here, we found that LE or de-glycyrrhizinated LE could effectively attenuate the Notch2 pathway in HG-cultured NRK-52E cells via multiple different mechanisms (Figure 8). These different mechanisms include suppressing HG-stimulated upregulation of cleaved NICD2, Jagged-1, Delta-like 1, nicastrin, and MAML-1, as well as blocking NICD2-mediated downstream signaling (Figure 8).

Although GC has been generally considered as the major bioactive component of licorice, our experiments did not support a significant role for GC in regulating EMT or Notch signaling in renal tubular epithelial cells. Under our experimental conditions, GC at 100 ng/mL or 300 ng/mL exhibited only partial inhibitory activities against HG-induced EMT in NRK-52E cells; however, GC at 10 ng/mL or 30 ng/mL had no significant effect on HG-induced EMT in NRK-52E cells (Figure 3). Previously, Hou et al. [36] have ever shown that GC at an extremely high concentration (82 μg/mL or 100 μmol/L) could have a beneficial effect in preventing HG-induced injury in renal tubular epithelial cells. Since the maximal amounts of GC used in our studies was only 0.3 μg/mL, the impact of GC at higher concentrations in regulating HG-induced EMT (or cell injury) in renal tubular epithelial cells remains to be explored. In addition to GC, licorice contains numerous potential bioactive components, including more than 20 triterpenoids and nearly 300 flavonoids [17,18,21]. De-glycyrrhizinated licorice has currently been manufactured to avoid the potential adverse effects of GC [22]. Similar to LE, de-glycyrrhizinated LE effectively inhibited HG-induced EMT in NRK-52E cells. Currently, the key bioactive components within LE, which inhibit HG-induced EMT in NRK-52E cells, are still unknown. Due to the fact that LE or de-glycyrrhizinated LE could suppress the Notch2 signaling cascade through different ways in HG-cultured NRK-52E cells, different bioactive components within LE or de-glycyrrhizinated LE may be involved in the suppressive function. When we went back through all previously reported studies, we noticed that several components from licorice, including licochalcone A (LCA), licochalcone E (LCE), liquiritigenin (LTG), glabridin (GLD), glabrol and amorfrutins, could potentially have anti-diabetic effect [37,38,39,40,41]. Despite their anti-diabetic potential, it still remains unclear whether these components could specifically modulate renal tubular EMT (or Notch2 pathway) under diabetic conditions. Further understanding the effects of these individual components, either alone or in combinations, on HG-stimulated EMT (or Notch2 activation) may provide helpful information to guide new licorice preparations for the treatment of renal tubulointerstitial fibrosis in diabetes.

In summary, we demonstrate here that the Notch2 signaling activation is critically involved in the development of tubular EMT under HG conditions, and find that LE or de-glycyrrhizinated LE can suppress tubular EMT by attenuating the Notch2 signaling pathway. We therefore suggest that LE or de-glycyrrhizinated LE could be potential therapeutic agents against the progression of renal tubulointerstitial fibrosis in diabetes. For future research, the most important direction could be to demonstrate whether LE or de-glycyrrhzinated LE indeed works to decrease diabetic renal fibrosis in animal models.
